# C_59_N Peapods Sensing the Temperature

**DOI:** 10.3390/s130100966

**Published:** 2013-01-15

**Authors:** Yongfeng Li, Toshiro Kaneko, Rikizo Hatakeyama

**Affiliations:** 1 State Key Laboratory of Heavy Oil Processing, College of Chemical Engineering, China University of Petroleum, Changping District, Beijing 102249, China; 2 Department of Electronic Engineering, Tohoku University, Sendai 980-8579, Japan

**Keywords:** azafullerenes, carbon nanotubes, encapsulation, photoresponse

## Abstract

We report the novel photoresponse of nanodevices made from azafullerene (C_59_N)-encapsulated single-walled carbon nanotubes (C_59_N@SWNTs), so called peapods. The photoconducting properties of a C_59_N@SWNT are measured over a temperature range of 10 to 300 K under a field-effect transistor configuration. It is found that the photosensitivity of C_59_N@SWNTs depends very sensitively on the temperature, making them an attractive candidate as a component of nanothermometers covering a wide temperature range. Our results indicate that it is possible to read the temperature by monitoring the optoelectronics signal of C_59_N@SWNTs. In particular, sensing low temperatures would become more convenient and easy by giving a simple light pulse.

## 1. Introduction

Sensing over a wide variety of temperature ranges is a challenging task, and most of the traditional thermometers are usually bulky, too large, and expensive. It has been demonstrated previously that the encapsulation of gallium inside a carbon nanotube is useful for a precise temperature measurement [1], but detecting low temperatures below 50 °C is limited due to the solidification of gallium. Recently, the research on single-walled carbon nanotubes (SWNTs) has uncovered numerous intriguing electronic and optical properties that could be used to develop new “smart” SWNT-based device systems with more degrees of freedom in performance by appropriate modification owing to their unique electrical and structural properties. In particular, compared with the case of multi-walled carbon nanotubes, the conductance change in SWNTs after modification with molecules or atoms makes them a potential material for sensor development [2–6]. In addition, one-dimensional structures and nanometer-range diameters of SWNTs make it possible to develop a high-density nanosensor array within a limited space. For example, SWNTs based on field-effect transistor configurations have shown unique or enhanced sensitivity toward gaseous species attracted much attention [7–10]. However, up to now there are few reports about the sensor performance of SWNTs which is related to the temperature sensor.

In this work, it is the first time for us to develop a temperature sensor based on the configuration of field-effect transistor (FET) with a C_59_N@SWNT as current channel. Our findings indicate that the photoinduced current of C_59_N@SWNTs depends sensitively on the temperature, which makes them a promising candidate as a component of nanothermometers.

## 2. Experimental Section

The azafullerene C_59_N was synthesized by a nitrogen plasma ion-irradiation method, which was confirmed by using a laser-desorption time-of-flight mass spectrometer (LD-TOF-MS, Shimadzu AXIMA-CFR+). The encapsulation of azafullerene C_59_N inside SWNTs is realized by either a vapor reaction method or a plasma ion-irradiation method [11]. In the case of the vapor reaction method, the purified SWNTs together with C_59_N azafullerene powders are first sealed in a glass tube under vacuum ∼10^−5^ Torr. Then the sealed glass tube is heated at 420 °C for 48 h to encapsulate C_59_N in SWNTs. Raw samples are obtained after the above process, and then purified via a washing process in toluene to remove the excess C_59_N attached to the surface of SWNTs. The purified peapods are examined in detail by Field Emission Transmission Electron Microscopy (FE-TEM, Hitachi HF-2000 and quanta 200F) operated at 200 kV.

The electronic transport properties of C_59_N@SWNTs are investigated by using them as the current channels of field-effect transistor (FET) devices [11,12]. First the C_59_N@SWNTs sample is ultrasonically dispersed in N,N-dimethylformamide and then spincoated onto FET substrates, each of which consists of Au source-drain electrodes with a channel length of 500 nm on a SiO_2_ insulating layer. A heavily doped Si substrate serves as a backgate. Photoinduced transport measurements are performed in the temperature range of 10–300 K under vacuum conditions on a semiconductor parameter analyzer (Agilent 4155C). Light illumination is carried out by a 150 W Xe lamp (LSX-2501) equipped with a monochromator to select the incident excitation wavelength (390∼1,100 nm). The light illumination intensity is less than 50 mW/cm^2^.

## 3. Results and Discussion

**Figure 1.**
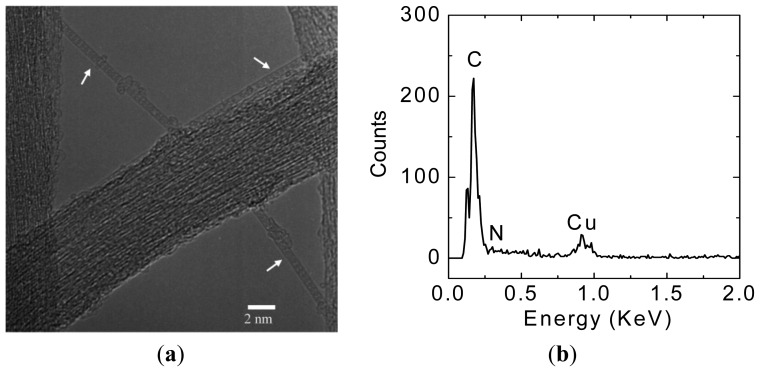
(**a**) A TEM image of C_59_N@SWNTs. (**b**) EDX of C_59_N@SWNTs.

**Figure 2.**
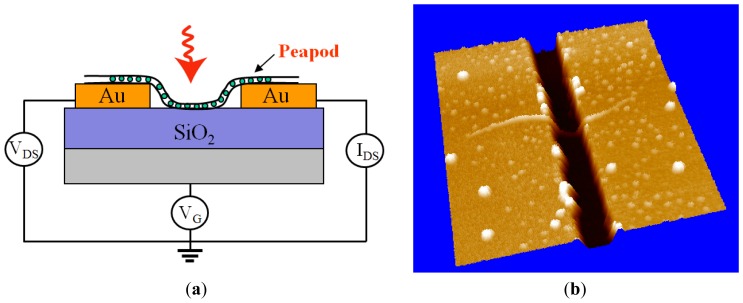
(**a**) Schematic illustration of FET configuration with a SWNT as current channel. (**b**) An AFM image of C_59_N@SWNT-FET.

A TEM observation result of C_59_N@SWNTs is shown in Figure 1(a), which indicates that C_59_N molecules with spherical symmetry are filled into both individual and bundled SWNTs. In comparison with an empty pristine SWNT, C_59_N molecules with spherical symmetry are clearly observed in the individual SWNTs, forming a one-dimensional chain-like structure inside the SWNTs, as illustrated by arrows in the TEM image. Therefore, the TEM observation provides strong evidence that C_59_N molecules are encapsulated inside SWNTs. The energy dispersive X-ray analysis is also used to distinguish the elements in the C_59_N@SWNTs during TEM measurements, as indicated in Figure 1(b). However, only C and Cu originated from the TEM copper grid are detected, and there is no signal of N found in the spectrum due to very small amount of N from C_59_N. A schematic diagram of FET device with a C_59_N@SWNT as current channel is illustrated in Figure 2(a), and its corresponding AFM image of the FET device is shown in Figure 2(b), in which a C_59_N@SWNT contacting two Au electrodes is clearly observed, which well confirms that an individual C_59_N@SWNT indeed plays the role of the current channel with gap width of 500 nm.

**Figure 3.**
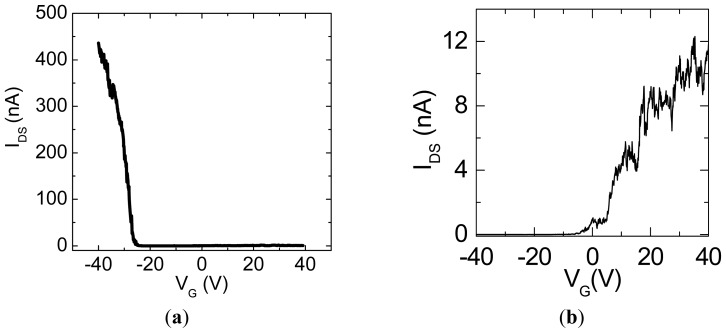
(**a**) *I_DS_*-*V_G_* curve measured with *V_DS_* = 1 V for a pristine SWNT-FET device. (**b**) *I_DS_*-*V_G_* curve measured with *V_DS_* = 1 V for a C_59_N@SWNT-FET device.

The transport properties of pristine semiconducting SWNTs are well known to exhibit the *p*-type behavior, as shown in Figure 3(a) [11], where a characteristic curve of source-drain current *I*_DS_ *versus* gate voltage *V*_G_ is described for source-drain voltage *V*_DS_ = 1 V. Figure 3(b) presents the transport property of C_59_N@SWNTs where the *I*_DS_-*V*_G_ curve is measured at *V*_DS_ = 1 V. In contrast, the transport property of C_59_N@SWNTs drastically changes to an *n*-type semiconductor. This *n*-type characteristic is attributed to the charge transfer between C_59_N and local parts of SWNTs [11–13]. It is found that such azafullerene-induced characteristics have been observed in many independent SWNTs devices and they have good reproducibility under measurements performed with different source-drain voltages, which has been confirmed in our previous work [11].

**Figure 4.**
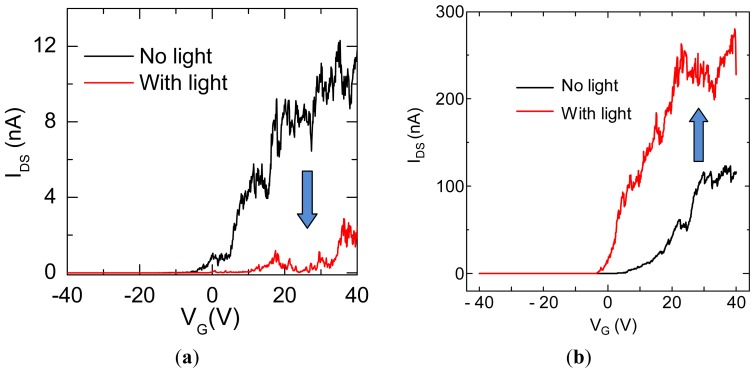
(**a**) *I_DS_-V_G_* characteristics (*V_DS_* = 1 V) of a C_59_N@SWNT-FET device measured without and with light (400 nm) illumination at room temperature 300 K. (**b**) *I_DS_-V_G_* characteristics (*V_DS_* = 0.5 V) of a C_59_N@SWNT-FET device measured without and with light (400 nm) illumination at low temperature of 10 K.

**Figure 5.**
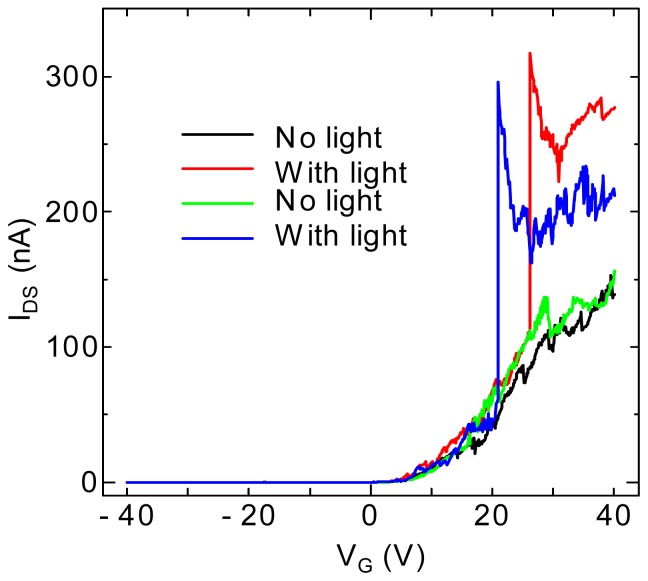
*I*_DS_-*V*_G_ characteristics (*V_DS_* = 0.5 V) measured at 10 K for an *n*-type C_59_N@SWNT with a light pulse (400 nm) at *V_G_* = 21 V and 26 V.

**Figure 6.**
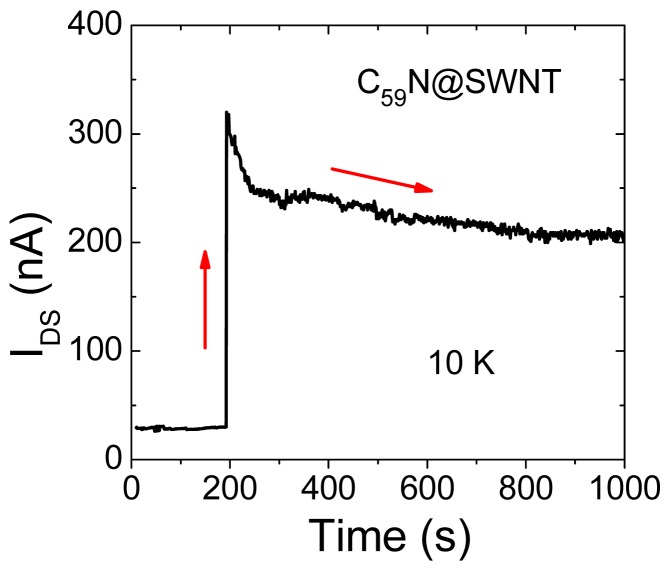
*I*_DS_ measured as a function of time at 10 K under exposure of a light pulse (400 nm) for a C_59_N@SWNT-FET device with *V*_DS_ = 0.5 V.

Under light illumination, it is noticed that the photoresponse of transfer characteristics for C_59_N@SWNT-FET is strikingly different at different temperatures. Figure 4 shows the transfer curves of a C_59_N peapod FET device, in which the *I*_DS_-*V*_G_ curves are recorded for the C_59_N@SWNT-FET in both dark and upon 400 nm light illumination for the two temperatures at 300 K and 10 K, respectively. Obviously, the prominent response of the device at room temperature to light is the decrease of transconductance, and the light irradiation results in ∼95% decrease in conductance (Figure 4(a)), which has been mentioned in our previous report [12]. However, at low temperatures such as 10 K, a different photoresponse phenomenon is observed in the transport property of C_59_N peapod FET device under light illumination, as shown in Figure 4(b), and the source-drain current displays a several times increase under the same light illumination, which is the exactly opposite phenomenon to that observed at 300 K. It is necessary to mention that such a phenomenon has never been observed in pristine SWNT FET devices. This finding indicates that the response of transport properties of C_59_N peapod FET device significantly depends on the variations of the temperature. On the other hand, the above interesting phenomenon implies a clear photoinduced electron transfer process. To further investigate the photoswitching characteristics at low temperatures, we have further exposed the device to the light pulse (1 s) during sweeping the *I*_DS_-*V*_G_ curves at 10 K, as seen in Figure 5. As the gate voltage is continually swept (with sweeping speed ∼1.4 V/s), the current at *V*_G_ = 21 V shows a sudden increase and the current value is about 6 times larger than its original one. After scanning to the high positive gate voltage *V*_G_ = 40 V, the measured *I*_DS_ is two times larger compared with the case of no light illumination. Again, when the 400 nm light pulse exposure is given at *V*_G_ = 26 V during *I*_DS_-*V*_G_ sweeping, the similar sharp increase of current is observed, suggesting that such an effect of current increase is fully reproducible by exposure to the light pulse and disappears without light illumination, demonstrating the complete restoration of photoswitching effect. The results confirm well that the C_59_N@SWNT FET device also exhibits an ultra fast response (on the level of millisecond) to the pulsed light, and the measured current is drastically enhanced under instantaneous UV illumination (400 nm, 1 s), which is entirely consistent with the result observed in Figure 4(b). Moreover, the *I*_DS_ measured (*V*_G_ = 20, *V*_DS_ = 0.5 V) as a function of time at 10 K under exposure of a light pulse for a C_59_N@SWNT-FET device is shown in Figure 6, suggesting strong evidence for the great increase in current under the light pulse. This finding is well consistent with the measured results in Figure 5, which is different from that observed at room temperature.

**Figure 7.**
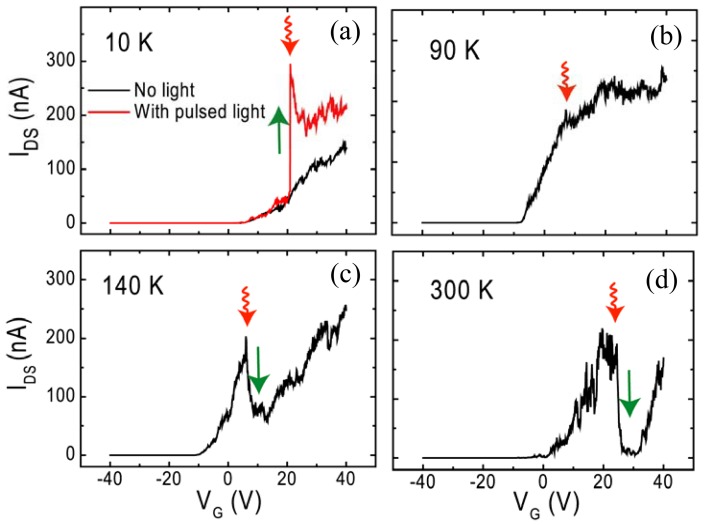
Photoinduced currents observed during tracing the *I*_DS_-*V*_G_ curves upon a light pulse at (**a**) 10 K, (**b**) 90 K, (**c**) 140 and (**d**) 300 K, respectively.

In order to understand the effect of temperatures, we have further measured photoinduced characteristics of current *vs.* light pulse at different temperatures, as indicated in Figure 7. Interestingly, the current increase is found to depend inversely on the temperature, and becomes gradually negligible when the temperature is increased from 10 to 90 K. As the temperature is further increased to 140 K, a clear negative photocurrent, *i.e.*, a decrease in current is observed, as seen in Figure 7(c), upon pulsed light illumination. Up to 300 K, a significant decrease of current upon the light pulse is observed, just in agreement with the result in Figure 4(a). Therefore, the above results suggest that it is possible to read the temperature by monitoring the optoelectronics signal of C_59_N@SWNT-FET.

**Figure 8.**
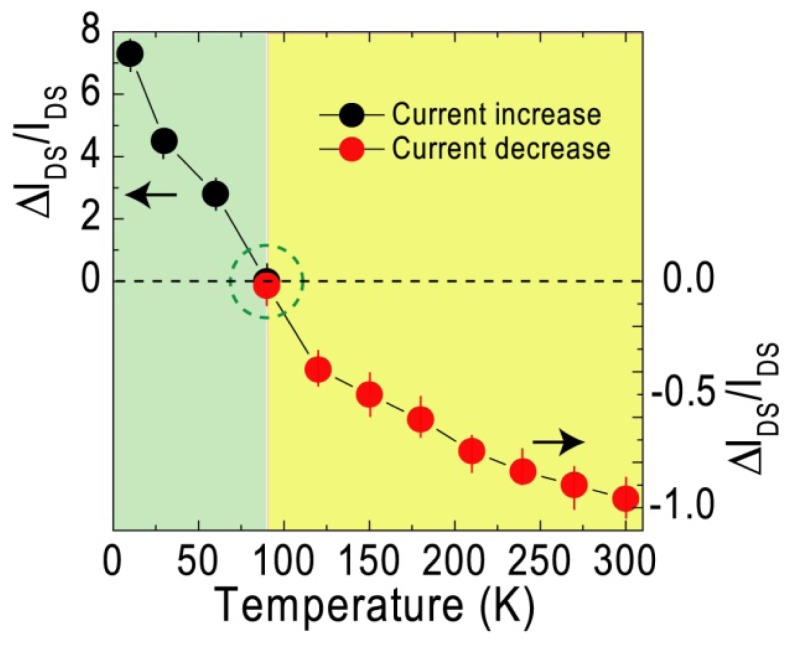
Variation of photoinduced current (Δ*I*_DS_/*I*_DS_) measured with temperature under exposure of a light pulse (400 nm).

Figure 8 presents the ratio of the changed current (Δ*I*_DS_) caused by instantaneous light illumination to the original current (Δ*I*_DS_/*I*_DS_) as a function of temperature in the range of 10–300 K. A variation of photoinduced current *vs.* temperature indicates that when the temperature is decreased and increased from 90 K in the range of 10–300 K, the positive and negative photocurrents rise, respectively. In other words, the photocurrent is found to depend inversely on the temperature, and it becomes gradually negligible when the temperature is increased from 10 to 90 K. As the temperature is further increased from 90 K to 300 K, a negative photocurrent is observed upon pulsed light illumination. This finding reveals that it is possible to read the temperature by monitoring the optoelectronics signal of C_59_N@SWNTs. In particular, sensing low temperatures would become more convenient and easy by giving a simple light pulse. In order to understand the photoswitching mechanism, we have further measured the transport properties of C_60_ fullerenes encapsulated SWNT (C_60_@SWNT) under the same experimental conditions of light illumination. There is no big change in the conductance under light illumination when the sample was measured at room and low temperatures, implying that the azafullerene is responsible for the decrease of conduction. According to our previous work [11–14], the *n*-type transport behavior of C_59_N@SWNT is considered to be due to the charge transfer from monomer C_59_N to SWNT by the weak C-C bonding since the azafullerenes C_59_N can easily lose or gain electrons through regioselective reactions. According to theoretical calculations [15], such bonding can easily undergo homolysis under photolysis or thermolysis conditions, resulting in the formation of azafullerenenyl radical C_59_N^•^. When the light energy is higher than the bonding energy, the thermolysis of the bond will lead to the stop of charge transfer, leading to a decrease in current at room temperature. To confirm this, we have also measured the transport behavior of C_59_N@SWNT at high temperatures (supporting information), and find that the current becomes unstable when the temperature reaches 400 K which indicates that the bond between C_59_N and SWNT will break due to the high system temperature or light absorption. However, the thermal effect is reduced in the low temperature environment, as a result, the weak bonding between C_59_N and SWNT may remain linked under light illumination. On the other hand, it was reported that the C_59_N^+^ exhibits distinguishing absorption spectral features in absorption spectra in the range of 1–3 eV [16]. The light energy exerted on the C_59_N@SWNT-FET device is in the range of 1.24–3.1 eV. Therefore, the multiplication effect cannot be ruled out since the quantum efficiency will be greatly enhanced at low temperatures. The above phenomenon might lead to the increase of photoinduced current at low temperatures although the exact mechanism is still unclear at this stage.

## 4. Conclusions

In summary, we have measured the transport properties of azafullerene peapods which act as the channels of FET devices, both in the dark and upon light illumination. It is found that the photosensitivity of C_59_N@SWNT-FET device depends very sensitively on the temperature compared with that of pristine SWNTs. At low temperatures the currents measured with sweeping gate voltages exhibit a remarkable increase, and the variation value of current is significantly dependent on the temperatures. However, the measured current shows a significant decrease when the device is illuminated with light at high temperatures, which is in sharp contrast to the low-temperature photoresponse. Such nanopeapod devices with distinguishing photoinduced properties are expected to have promising applications as a component of nanothermometers covering a wide temperature range.
